# People’s preferences for self‐management support

**DOI:** 10.1111/1475-6773.13635

**Published:** 2021-02-25

**Authors:** Cynthia P. Iglesias Urrutia, Seda Erdem, Yvonne F. Birks, Stephanie J. C. Taylor, Gerry Richardson, Peter Bower, Bernard van den Berg, Andrea Manca

**Affiliations:** ^1^ Department of Health Sciences University of York York UK; ^2^ Stirling Management School University of Stirling Stirling UK; ^3^ Social Policy Research Unit University of York York UK; ^4^ Barts and The London School of Medicine and Dentistry Queen Mary University of London (QMUL) London UK; ^5^ Centre for Health Economics University of York York UK; ^6^ Centre for Primary Care Institute of Population Health University of Manchester Manchester UK; ^7^ Department of Health Sciences VU University Amsterdam Amsterdam The Netherlands

**Keywords:** long‐term conditions, mixed methods, person‐centered health care, preferences, self‐management support interventions

## Abstract

**Objective:**

To identify and assess the preferences of people with long‐term health conditions toward generalizable characteristics of self‐management support interventions, with the objective to inform the design of more person‐centered support services.

**Data Sources:**

Primary qualitative and quantitative data collected on a representative sample of individuals with at least one of the fifteen most prevalent long‐term conditions in the UK.

**Study Design:**

Targeted literature review followed by a series of one‐to‐one qualitative semistructured interviews and a large‐scale discrete choice experiment.

**Data Collection:**

Digital recording of one‐to‐one qualitative interviews, one‐to‐one cognitive interviews, and a series of online quantitative surveys, including two best‐worst scaling and one discrete choice experiment, with individuals with long‐term conditions.

**Principal Findings:**

On average, patients preferred a self‐management support intervention that (a) discusses the options available to the patient and make her choose, (b) is individual‐based, (c) face to face (d) with doctor or nurse, (e) at the GP practice, (f) sessions shorter than 1 hour, and (g) occurring annually for two‐third of the sample and monthly for the rest. We found heterogeneity in preferences via three latent classes, with class sizes of 41% (C1), 30% (C2), and 29% (C3). The individuals’ gender [*P* < 0.05(C1), *P* < 0.01(C3)], age [*P* < 0.05(C1), *P* < 0.05(C2)], type of long‐term condition [*P* < 0.05(C1), *P* < 0.01(C3)], and presence of comorbidity [*P* < 0.01(C1), *P* < 0.01(C3), *P* < 0.01(C3)] were able to characterize differences between these latent classes and help understand the heterogeneity of preferences toward the above mentioned features of self‐management support interventions. These findings were then used to profile individuals into different preference groups, for each of whom the most desirable form of self‐management support, one that was more likely to be adopted by the recipient, could be designed.

**Conclusions:**

We identified several factors that could be used to inform a more nuanced self‐management support service design and provision that take into account the recipient's characteristics and preferences.

**What This Study Adds**

**What is already known on this topic**

- Recent work found that self‐management support—aimed at behavioral change and supporting self‐efficacy—can have a positive impact on people's clinical symptoms, attitudes, quality of life, and patterns of health care resource use.
- Evidence as to what are the best strategies to support behavior change and self‐efficacy is limited and uncertainty remains regarding how people prefer to be supported.

**What this study adds**

- We used mixed methods to study the preferences of a large UK sample of individuals with long‐term conditions toward key characteristics of self‐management support.
- By taking into account the recipient's characteristics and their preferences toward specific features of self‐management support interventions, we profiled individuals as belonging to one of three preference classes.
- We showed how information about the preference structure in each of these classes can be used to design a more person‐centered self‐management support service, effectively moving away from a “one‐size‐fits‐all” provision.

## 1 INTRODUCTION

Noncommunicable long‐term health conditions (LTCs) in England account for approximately 50% of all primary care visits, 64% of all outpatient appointments, and over 70% of all hospital inpatient stays.^1^ This means that around 70% of the total health care spend in the National Health Service (NHS) can be attributed to caring for people with LTCs.^1^ A recent report from RAND estimated that the scenario is no different in the United States where, in 2014, 60% of the population had at least one LTC, 42% had multiple LTCs, and among the individuals aged 65+, the prevalence of multiple LTCs was 81%.^2^

Interventions where patients take on more responsibility for managing their health, giving them a more central role, and choice, regarding their everyday care have been widely recommended in an attempt to change the traditional patient‐doctor dyad and relieve the pressure on the health care system.^3^, ^4^, ^5^, ^6^ The evidence regarding the ability of self‐management interventions to reduce health care resource use and improve quality of life is encouraging but inconsistent.^7^, ^8^, ^9^ One reason for this is that strategies to *support* good self‐management are often missing from the equation, despite being inseparable from the high‐quality care that people with LTCs should receive.^10^

*Self‐management support* (SMS) interventions are a crucial component in the pursuit of an efficient model of health care provision.^7^, ^11^, ^12^, ^13^ Recent evidence reviews^10^, ^14^ found that SMS—aimed at behavioral change and supporting self‐efficacy—can have a positive impact on people's clinical symptoms, attitudes, quality of life, and patterns of health care resource use. At this stage however, with some important exceptions, evidence as to what are the best strategies to support behavior change and self‐efficacy is limited and uncertainty remains regarding how people prefer to be supported and how these preferences may vary between individuals.^10^, ^14^

We argue that to develop an effective SMS intervention for individuals with LTCs requires understanding (a) what characteristics of these interventions matter to patients, (b) the value that individuals place on these features ^15^ , (c) what trade‐offs recipients are prepared to make between characteristics that may define different SMS interventions, and to use this information to (d) tailor SMS provision to the characteristics and needs of the recipient. This requires providers to move away from a *one‐size‐fits‐all* approach to SMS commissioning and embrace a more flexible person‐centered delivery of these interventions.

This study aimed to quantify the preferences that people with LTCs have toward SMS interventions. Rather than focus on *specific* SMS interventions for *a given* LTC, we posit the former in terms of a series of features *generalizable* (across interventions) that may describe them and study these in the population of individuals diagnosed with one or more of the 15 most prevalent LTCs according to the NHS Quality and Outcomes Framework statistics.^1^

## 2 METHODS

We adopted a multilayered mixed‐methods research strategy, structured into four self‐contained, sequential, and interdependent phases (Figure S1A). *Phase 1* developed an initial classification of SMS interventions based on the findings of a literature review conducted to identify their key *generalizable* characteristics. *Phase 2* comprised a series of in‐depth qualitative one‐to‐one interviews with people with LTCs to test, refine, and finalize the classification of SMS intervention characteristics that mattered to responders. The resulting classification informed *Phase 3*, which involved designing, piloting, and conducting a large‐scale choice‐based online quantitative survey to elicit the preferences people with LTCs have toward key generalizable features of SMS interventions and to assess the trade‐offs individuals are prepared to make between these characteristics. The results of the choice‐based survey were used in *Phase 4* to review and update the initial classification of generalizable characteristics of SMS interventions. The methods used as part of each of the study phases are described in turn.

### 2.1 Phase 1: Initial classification of generalizable characteristics of SMS interventions

We carried out an analysis of two recent literature reviews of SMS interventions.^10^, ^14^ Members of our research team extracted an initial list of SMS intervention characteristics resulting from the report published by de Silva,^14^ which were combined and further elaborated to generate an initial classification. To verify the relevance of the identified characteristics in the context of the wider literature, we mapped these against the PRISMS’ taxonomy of SMS interventions,^16^ a process further validated in a face‐to‐face meeting with PRISMS’ team researchers and subsequently by members of our scientific advisory group.

### 2.2 Phase 2: Qualitative interviews

We conducted a series of semistructured one‐to‐one interviews^17^ at either the participant's home or by phone with a sample of individuals recruited for us by the survey research company Ipsos MORI (more information about their online sample can be found here https://www.ipsos.com/ipsos-mori/en-uk/survey-methods-ipsos-mori#notes). Each interview lasted 60‐90 minutes and was structured around a schedule of topics and prompts that aimed to identify important SMS characteristics—that is, attributes for the discrete choice experiment (DCE)—and their associated dimensions (ie, levels for the DCE) that may have been missed in Phase 1. The interviews were fully transcribed and analyzed using the *framework approach* ^18^ to describe the views on SMS strategies represented within the participant sample. The topic guide (available on request) was informed by the classification produced in Phase 1.

The framework approach is an established and transparent methodology, well suited to qualitative analysis in the context of a multidisciplinary project. It enables development of responses to a priori research questions while remaining open to unanticipated perspectives of participants. Both deductive and inductive logic can be employed to reduce and synthesize data in five steps. First familiarization was achieved by repeated reading of the transcripts as they were generated. Data coded descriptively to reflect content was then allocated to an initial frame, in which cells represented data sources by research question (with multiple allocations permitted), or “other” (data not relevant to research questions). A process of constant comparison is then used to iteratively develop case‐ and question‐based summaries of data. Patterns and exceptions are identified with similar data grouped and descriptively labeled to produce subcategories.

### 2.3 Phase 3: Designing, piloting, and conducting a choice‐based online survey

#### 2.3.1 Attributes and attribute levels

The results of Phases 1 and 2 yielded a list of potential attributes that could be used to characterize SMS interventions. To understand how patients ranked these attributes in terms of importance to them, we conducted two sets of best‐worst scaling (BWS) exercises,^19^ each with a sample of 200 respondents. This is an important step in the design of a DCE since the inclusion of more attributes than what are strictly necessary in a given context can result in a survey that is too complex for respondents, potentially compromising the preferences elicitation exercise. Details of the BWS survey design and results are available on request from the author for correspondence. To verify the feasibility, as well as validate the pilot DCE survey content and framing, we conducted a series of one‐to‐one cognitive interviews (also known as think‐aloud sessions) with patients.

#### 2.3.2 Choice‐based online survey

Our survey began with a series of screening questions to identify eligible respondents. Individuals with more than one LTC (ie, multimorbidities) were asked to indicate which of these was the most burdensome for them. Participants were provided information on the study, a consent form, examples of SMS interventions, instructions on how to complete a 10‐question choice‐based task, and questions about their health and attitudes to health. Our survey concluded asking respondents to (a) rank in order of importance four nonmutually exclusive aims of a SMS intervention that emerged from Phase 2 (ie, to improve: confidence, medical knowledge and skills, lifestyle and well‐being, or to understand triggers and motivations,); and (b) provide sociodemographics, current travel time, means of transport to primary care physicians (ie, GPs) and local hospitals, and views on the survey. The study received approval by the Research Governance Committee of the Department of Health Sciences at the University of York, UK.

#### 2.3.3 Choice task

To design a 10‐question choice‐based task for our online survey we used one of the stated preferences techniques, that is, DCE. Choice questions involved asking respondents to indicate which of two hypothetical alternative SMS options, defined by a combination of their generalizable characteristics (ie, attributes and attributes levels), they preferred. After each DCE question, respondents were asked to indicate—if the opportunity arose in real life—whether they would in fact use a SMS intervention with the characteristics of the option they had selected.

#### 2.3.4 DCE experimental design

The pilot and final DCE used a partial‐profile experimental design created using the Sawtooth Software.^20^, ^21^, ^22^, ^23^ In particular, our survey presented participants with five attributes, two of which appeared in each of the 10 questions in our choice‐based question task, with the remaining three attributes varied with every choice set, while ensuring a nearly orthogonal experimental design with frequency balance and balanced overlap between attributes and levels. Figure S2A reports a sample choice task.

#### 2.3.5 Background questions

Each participant was asked to report their gender, age, ethnicity, employment status, and frequency of NHS use. Furthermore, respondents were invited to complete the EQ‐5D‐3L^24^ and the Patient Activation Measure (PAM)^25^, ^26^ questionnaires. The former is a generic measure of health‐related quality of life that classifies 243 different health states (plus dead and unconscious) through five dimensions of health (mobility, self‐care, usual activities, pain/discomfort, and anxiety/depression) each with three levels of severity.^27^ The PAM is a unidimensional scale which can be used to classify respondents in one of four levels of activation, capturing the extent to which people feel engaged and confident in taking care of their condition.^25^

#### 2.3.6 Sampling strategy and process

Our recruitment strategy employed sampling quotas that controlled for age, gender, and estimates of disease prevalence—obtained from the UK Office for National Statistics (ONS) General Lifestyle Survey (GLS)^28^—and combined these with figures from the Quality and Outcomes Framework (QOF) in England. Members of Ipsos MORI online sample were sent an email with a Web link to our survey. To verify the quality of our sampling framework, we administered the survey in three separate waves recruiting 1150, 2300, and 1164 respondents, respectively. This allowed us to (a) enhance representativeness by adjusting (where necessary) the sampling strategy between waves; (b) prevent systematic over/under‐sampling by age and gender; and (c) conduct interim data quality checks (eg, for excessively short completion time). The overall data collection took four weeks.

#### 2.3.7 Choice data analysis

Responses to the DCE survey were analyzed using a latent class model (LCM) to allow for preferences’ heterogeneity across respondents to be quantified and explained.^29^

The underlying theory of the LCM posits that individuals’ choice behavior depends on both observable attributes and unobserved latent factors. The model allocates respondents into a set of preference classes, which are not observable by the analyst. Individuals within each class are assumed to share the same preferences, but differences exist between classes.

As there is no universally accepted criterion for choosing the optimum number of latent preference classes, we considered a number of factors in deciding the class number. We analyzed the choice data using LCMs with 1 to 5 classes and found that the model with three preference classes was the best model explaining choices, according to the Bayesian information criterion and Akaike's information criterion, log‐likelihood values, and model parsimony considerations. The statistical analysis was performed using Latent GOLD Syntax 4.5.^30^

### 2.4 Phase 4: Revision of the classification of generalizable characteristics of SMS interventions

This phase involved returning to our initial classification of generalizable characteristics of SMS interventions to update it with the results of the main online choice‐based survey.

## 3 RESULTS

### 3.1 Phase 1: Initial classification of generalizable characteristics of SMS interventions

The initial classification exercise yielded six broad concepts: target *audience*, target *area of support*, *focus* of the support intervention, *who* is the individual *the patient interacts with* when receiving the support intervention, *mode of delivery* of the intervention, and features associated with the *time investment* the patient needs to make with a particular intervention. A brief description of these concepts is provided in Box S1A.

### 3.2 Phase 2: Qualitative Interviews

Ipsos MORI contacted 500 individuals from their online panel inviting them to complete a screening questionnaire about their sociodemographic, what LTC they were diagnosed with, if any, and whether they were willing to be contacted by our research team. Twenty‐four of the first 71 respondents that best reflected gender, age, ethnicity, and prevalence distribution were recruited and interviewed. The sample was mostly white British (three participants were from ethnic minorities), between 30 and 78 years old (average 61), 52% were female, and 46% of them had more than one LTC.

Participants articulated a number of ways in which SMS interventions could be structured, although views varied widely and no consistent patterns emerged. They were interested in knowing about their condition, and what treatment, management, and support options were available, even if not all of them could be provided by the NHS.

When asked how they would prefer SMS to be delivered—despite a number of prompts about more creative or novel methods—responses tended to stress face‐to‐face contact. Those who had engaged with support groups often felt that these lacked legitimacy when they were not led by qualified medical professionals. Participants were generally enthusiastic about a responsive and flexible approach to support which would allow them to get information when they needed it, perhaps by phone rather than face to face. There was little use of email, although some people indicated they would be happy to use it if they knew someone would respond.

When asked what SMS should provide, respondents were keen to gain information from reliable sources to guide their choices (ie, local GPs and—for unusual complex conditions—disease specialists were the people identified as best suited to providing up‐to‐date information). Less reference was made to specific skills such as monitoring disease or using equipment. Participants valued support which would increase motivation or self‐efficacy and confidence in relation to their condition. This was linked to better knowledge of the condition but also confidence in the SMS provided.

When asked what style of SMS they would prefer, respondents expressed inconsistent views. Within the same interview, respondents might express a desire for more or less directive support in relation to different aspects of their condition(s) depending on the nature of the specific activity or particular temporal context (eg, how long it lasted; how frequently it took place). Many respondents articulated the need to differentiate between support strategies where they are told how to live their life and those where they are given information and support to allow them to make choices for themselves.

Finally, when asked how technology contributed to SMS, participants reported that their use of technology was limited to monitoring blood pressure and blood sugar levels. Users of these monitors largely fell into two categories: highly motivated regular users who fed back their readings to professionals, and those who monitored their own condition and proactively addressed diet, lifestyle, and medication. A number of people had monitors they did not use. Some felt that there was no point in monitoring their own condition as primary care professionals did this.

### 3.3 Phase 3: Designing, piloting, and conducting a choice‐based survey

#### 3.3.1 Final DCE attributes and attributes levels

Phases 1 and 2 resulted in an initial identification of 11 generalizable attributes of SMS interventions and 38 attribute levels (Table S1A). Findings from the BWS exercises identified that seven of the initial 11 attributes contributed to the choice process with relatively similar importance (Table 1). These were used to design the choice‐based task in our online survey.

**TABLE 1:** Attributes, levels, and description of SMS interventions for DCE task questions

Attribute	Attribute levels	Description
How long it takes	Less than 30 min Between 30 min and 1 h More than an hour	Amount of time people would set aside to engage with a SMS activity
How often it happens	Weekly Monthly Every 3 months Annually	This refers to how frequently a SMS activity takes place
Who leads it	Doctor Nurse Other health care professional Non health care professional	Characterization of the person delivering the SMS activity based on qualifications and training in health care
Where it happens	At GP At Hospitals In Home At Community	Setting where the SMS takes place
Style of interaction	Discuss options with me and tells me what to do Discuss options with me and lets me choose	Communication style of the person delivering the SMS activity
Type of interaction	Individual Group	Individual versus group intervention/SMS activity
Degree of human contact	Face to face Telephone / online On your own	The way the SMS activity is delivered

#### 3.3.2 Choice‐based survey sample and sample characteristics

Ipsos MORI sent the link to our choice‐based online survey to 18 249 individuals, of whom 75% were not eligible to participate: 47% did not have any of the LTCs we intended to sample; 1% completed the survey in less than 4 minutes; 11% did not respond to screening questions or indicated that the only LTC they had experienced was obesity; and 16% abandoned the survey prematurely. The remaining 25% (N = 4614) completed the survey which yielded an overall response rate, among eligible individuals, of 62%.

The baseline characteristics of our sample are comparable to the figures reported in the most recent UK Census. A comparison of the distribution of LTCs in our sample against the expected prevalence rates in our target population (Table S2A) suggests that the former was fairly representative. Approximately 55% of our survey participants reported one LTC, while the remaining 45% reported varying degrees of multimorbidity (see Table S3A—for the list of most burdensome LTCs reported by our sample). Sociodemographic characteristics, self‐reported health status (measured using EQ‐5D), and activation levels (measured using PAM) in our sample are reported in Table 2.

**TABLE 2:** Baseline characteristics of the final sample for the DCE

	Percent	N
Gender		
Male	47%	2169
Female	53%	2445
Age (mean = 56, SD = 16.4)		
Less than 45	48%	2204
45‐64	46%	2123
65‐74	6%	272
More than 75	<1%	15
Ethnicity		
White British	89%	4127
White Irish	1%	49
White other	4%	171
Black or Black British‐Caribbean	<1%	23
Black or Black British‐African	<1%	19
Black or Black British‐other	<1%	4
Chinese	<1%	12
Asian or Asian British‐Indian	1%	51
Asian or Asian British‐Pakistani	<1%	18
Asian or Asian British‐Bangladeshi	<1%	6
Asian or Asian British‐other	<1%	14
Mixed ethnicity	<1%	39
Other ethnicity	<1%	21
Prefer not to say	1%	60
Employment status		
Student	2%	89
Full‐time employed	22%	1036
Part‐time employed	9%	418
Unemployed (looking for a job)	3%	120
Unemployed (not looking for a job)	4%	198
Unable to work (eg, too sick or ill to work)	12%	542
Self‐employed	5%	251
Retired	43%	1960
Use NHSs regularly	93%	4294
General health		
Poor	11%	528
Fair	38%	1737
Good	39%	1800
Very good	10%	485
Excellent	2%	64
EQ‐5D (Mobility)		
No problem	56%	2604
Some problem	43%	1989
Confined to bed	<1%	21
EQ‐5D (Self‐care)		
No problem	84%	3875
Some problem	15%	695
Unable to wash or dress	1%	44
EQ‐5D (Usual activities)		
No problem	53%	2437
Some problem	42%	1942
Unable to perform usual activities	5%	235
EQ‐5D (Pain/Discomfort)		
No problem	37%	1697
Some problem	52%	2409
Extreme pain/discomfort	11%	508
EQ‐5D (Anxiety/Depression)		
No problem	55%	2513
Some problem	37%	1717
Extremely anxious/depressed	8%	384
PAM levels		
Level 1—disengaged and overwhelmed	17%	796
Level 2—becoming aware, but still struggling	19%	852
Level 3—taking action	45%	2087
Level 4—maintaining behavior and pushing further	19%	859

Abbreviations: NHS, National Healthcare Service for England; PAM, patient activation measure; SD, standard deviation.

#### 3.3.3 Descriptive analysis of choice data

In the DCE survey, respondents were invited to select one of two SMS alternatives and to indicate whether they would use an intervention that had the characteristics of their selected option. Overall, we had 37 675 choice observations from 4455 respondents, of whom 75% said they would use the chosen option, 22% said they would not and approximately 3% were unsure.

#### 3.3.4 Estimation results

The results of the LCM (Table 3) show that preferences in our sample displayed a certain degree of heterogeneity, which led to three preference classes. On average, people in all three classes had a stronger preference for SMS interventions that (a) discuss the options available to the patient and let her choose, (b) are individual‐based, (c) carried out face to face, (d) at the GP practice, (e) led by a doctor or nurse, (f) take no longer than 1 hour per session, and (g) with sessions occurring annually (for two‐third of the sample).

**TABLE 3:** Latent class model estimation results (N = 4614)

	Beta	SE	Beta	SE	Beta	SE
	Class 1 (41%)		Class 2 (30%)		Class 3 (29%)	
Constants						
ASC(1)	0.76	0.08***	−1.14	0.07***	2.41	0.11***
ASC(2)	0.54	0.09***	−1.29	0.07***	2.35	0.11***
ASC(sq)	Baseline					
How long it takes						
Less than 30 min	0.09	0.03***	0.36	0.05***	0.74	0.05***
Between 30 min and 1 h	0.11	0.03***	0.32	0.05***	0.34	0.05***
More than 1 h	Baseline					
How often it happens						
Weekly	0.86	0.06***	−2.20	0.09***	−2.33	0.10***
Monthly	1.10	0.06***	−1.16	0.06***	−1.33	0.08***
Every 3 months	0.94	0.05***	−0.53	0.05***	−0.53	0.06***
Annually	Baseline					
Who leads it						
Doctor	0.48	0.05***	0.58	0.06***	0.49	0.07***
Nurse	0.55	0.05***	0.44	0.06***	0.13	0.07***
Non–health care	−0.78	0.05***	−0.68	0.07***	−0.60	0.08***
Other health care	Baseline					
Where it happens						
At GP	0.51	0.05***	0.65	0.06***	0.72	0.07***
At hospital	−0.09	0.05*	0.06	0.06	−0.14	0.08*
At home	0.17	0.05***	< 0.01	0.06	0.21	0.06***
Community center	Baseline					
Its style						
Discuss and makes decision for me	−0.22	0.03***	−0.27	0.05***	−0.23	0.05***
Discuss and lets me choose	Baseline					
Interaction type						
Individual based	1.03	0.03***	0.82	0.04***	1.06	0.05***
Group based	Baseline					
Degree of human contact						
Face to face	0.35	0.04***	0.43	0.05***	0.08	0.06
Phone/online	−0.45	0.04***	−0.08	0.06	0.01	0.06
On your own	Baseline					
Class membership						
Intercept	0.10	0.06*	−0.03	0.05	−0.08	0.06
Male (=1, yes)	−0.08	0.05*	−0.08	0.05	0.16	0.06***
Age (45‐64) (=1, yes)	−0.17	0.05***	0.16	0.05***	<0.01	0.06
Employed (=1, yes)	0.07	0.04*	−0.06	0.05	−0.01	0.06
Comorbidity (=1, yes)	0.40	0.05***	−0.14	0.05***	−0.26	0.06***
Log‐likelihood	−31 460					
AIC	63 042					
BIC	63 433					
N(parameters)	61					
N(respondents)	4455					

Note

Stars represent significant parameters at 1% (***), 5% (**), and 10% (*) levels.

Abbreviations: ACS, alternative specific constant; AIC, Akaike's information criterion; BIC, Bayesian information criterion; GP, general practitioner; SE, standard error.

However, there were several significant differences in preferences by class membership. Additionally, significant socioeconomic variables, such as gender, age, employment status, and comorbidity used in the class membership indicate that these segments are made up of different profiles of respondents.

In terms of differences in preferences, we observe that only class 2 (30% of our sample) included respondents who said that they would not use the chosen option. This class was characterized by individuals who were more likely to be unemployed, older than 44 years old, and with one LTC (Table 4). Individuals in class 2 had a strong preference for SMS interventions with a longer time interval between sessions. Individuals in class 1 (41% of our sample) were more likely to be employed, female, younger than 44 years old, and with more than one LTC. This group of individuals had a stronger preference for monthly SMS interventions at their GP surgery or in their home. In class 3 (29% of our sample), individuals were more likely to be men with one LTC, displaying a relatively strong preference for SMS interventions taking less than 30 minutes, no significant preference toward the degree of human contact, and less likely to accept hospital‐based SMS interventions.

**TABLE 4:** Describing preference heterogeneity

**Patient Class 1 (41%)**
*Less frequent SMS seekers at GP surgery, younger, employed females with multi‐comorbidity*
Prefer SMS taking less than 30 minutes the most Prefer monthly SMS the most Prefer doctors and nurses most, dislikes SMS led by non–health professionals Prefer SMS at GP surgery and in homes Prefer to engage in decisions about their health Prefer face‐to‐face SMS most and technology‐based SMS (eg, online/phone) least More likely to be female More likely to be employed More likely to have more than one conditions Tend to be younger than 45
**Patient Class 2 (30%)**
*Annual and short SMS seekers who have one chronic conditions, are more likely to be unemployed, older female individuals*
Prefer SMS taking less than 30 minutes the most Prefer annual SMS only Prefer doctors and nurses most, dislikes SMS led by non–health professionals Prefer GP surgery only Prefer to engage in decisions about their health Prefer face‐to‐face SMS only More likely to be female More likely to be unemployed More likely to have one condition Tend to be older than 45
**Patient Class 3 (29%)**
*Time focused men with one chronic condition and no significant preferences toward technology‐driven or face‐to‐face SMS*
Relatively strongly prefer less than 30 minute Annual SMS only Prefer doctors most, but nurses relatively less than other classes, and dislikes SMS led by non–health professionals Prefer GP surgery and home most, dislikes hospitals Prefer to engage in decisions about their health Do not have strong preferences toward technology‐based or face‐to‐face SMS More likely to be male More likely to have one condition No age and employment effect on class membership

Abbreviation: SMS: self‐management support.

To further explain the above three classes, we analyzed individual‐level class probabilities retrieved from the LC analysis with respect to individuals’ sociodemographic characteristics, self‐reported health status, and attitudes toward their health and ways of managing it (ie, PAM). Our results indicated that more than half (53%) of the respondents with chronic pain are more likely to belong to class 1. In terms of the severity of health conditions, we observed that the majority of the respondents were in the middle/stable stage of their conditions (73% of individuals with comorbidities, 82% of individuals with one condition).

The preference class characterized as more likely to have comorbidities (ie, class 1) also tended to be in a stable stage of their condition which may contribute to explain why individuals in this group preferred monthly or annual SMS, as opposed to weekly SMS.

When incorporating baseline patient‐reported outcomes in class characterization, we observed that individuals in class 1 and class 3 were less likely to have problems in the five dimensions of health considered by the EQ‐5D, unlike individuals in class 2.

Patient classes also showed some variation in the PAM. While overall the majority of individuals across the three classes was quite proactive and took action to improve their health (levels 3‐4), there was variability among those who had lower activation. Relative to class 3, respondents in class 1 and class 2 were more likely to have a lower level of activation (ie, PAM levels 1 and 2).

### 3.4 Phase 4: Revision of the classification of generalizable characteristics of SMS interventions

Armed with the results of the DCE, we went on to update the classification of generic components and dimensions of SMS interventions. This is reported in Table S5A.

## 4 DISCUSSION

To the best of our knowledge, this is the first study to use mixed methods to examine *generalizable* characteristics of SMS interventions in order to inform a broader policy and research agenda in this area. To do this, we elicited the preferences of a large sample of individuals with at least one of the 15 most prevalent LTCs in the UK.

Our findings confirm that a one‐size‐fits‐all approach is inefficient when making decisions regarding the provision of health care interventions to support patients’ self‐management efforts. As reported in the more general evaluation for decision making literature, ignoring heterogeneity within the patient population may have a profound impact in terms of population health forgone.^31^, ^32^ Therefore, ignoring the diversity of patient preferences toward the features of the interventions aimed to support their self‐management efforts has an impact on adherence, self‐efficacy, and health outcomes.^33^

A number of authors have recommended tailoring SMS strategies to patient profiles and preferences,^34^ with specific examples in heart failure and stroke.^35^, ^36^ There is some evidence that tailoring SMS strategies to patient activation levels can also help achieve greater health outcomes for patients.^37^ Our findings support these recommendations and demonstrate that preferences for the type and modality of SMS interventions they receive vary systematically across individuals. This might explain why some people do not value the support interventions that have been found to be—*on average*—the most (cost‐)effective strategy, very likely missing out on the opportunity to receive an alternative intervention that might better help them help themselves. For instance, previous evidence suggests that men display well‐defined preferences for and against specific SMS styles.^38^, ^39^ Similarly, a recent review found that the literature regarding the effectiveness of smartphone apps to support self‐management of asthma patients is sparse and inconclusive. The fact that patients in the study by Galdas et al^39^ complied with the use of these devices means that this technology has the potential to make SMS interventions more successful and accessible to some individuals.^40^ This result is consistent with our finding that men with a single LTC are more likely to adopt support strategies that are technology‐driven; an important finding since usually women are overrepresented in studies of (typically, group‐based) SMS interventions.^41^

While online sampling may be subject to selection bias (ie, not all people with LTC may be able or willing to participate in an online survey), the same is true for other forms of survey.^42^ We showed that it is relatively easy to use a sampling framework for selecting choice‐based surveys' participants to ensure a closer representativeness of the sample to the target population. It could be argued that most studies recruit from a pool of patients who may have already signed up to groups and displayed good adherence. The fact that a large proportion of our sample was SMS naive gave us a valuable opportunity to elicit and reflect the preferences of this group. In this sense, our findings may be useful to inform the design of SMS interventions more likely to be appealing to a SMS‐naive population. For instance, our seemingly conflicting result (with published literature in the area of SMS interventions), that on average people dislike group‐based SMS interventions, might be a reflection that group‐based SMS interventions are more likely to be unappealing to SMS‐naive populations.

The relevance of our study findings is not limited to the UK. Self‐management support is widely endorsed by the CDC (https://www.cdc.gov/learnmorefeelbetter/index.htm), whose strategy and research gaps in this area have been described recently by Brady *et al* ^43^ As importantly, our work helps address three of the seven strategic directions and sample actions (ie, 2, 3, and 4) identified by the consensus group that developed the International Chronic Condition Self‐Management Support Framework.^44^

There are a number of interesting policy implications we can draw from our results which are worth mentioning.

First, our results strongly suggest that SMS ought to be incorporated in routine primary care visits as respondents seem to like the idea of brief one‐to‐one interventions, and routine primary care is potentially the optimum place to deliver SMS as found by Kennedy et al^45^ Viable models to deliver this service however may be difficult to generate, and their provision may require significant upfront costs. While alternative delivery models may exist, the strong preferences displayed by our sample provide some food for thought for commissioners and service providers. The challenge is therefore to design a portfolio of SMS interventions that meets patients’ preferences while remaining effective, cost‐effective, and affordable.

Second, on average, respondents in our sample prefer shorter and less frequent SMS activities, delivered by a doctor or nurse. This is promising, as a recent systematic review of characteristics of SMS courses for people with painful LTCs found that a course of less than eight weeks was as effective as longer courses.^46^ The fact that—on average—people in our sample preferred one‐to‐one sessions at a GP practice may seem to contradict Carnes et al^46^ who found that individual and group interventions to be equally effective; however, the two studies looked at different outcomes. People's preference for GP practice one‐to‐one sessions may also appear to contradict existing qualitative research that suggests some individuals like group interventions and the opportunity to meet others with the same condition.^10^ As mentioned previously, the reason for this may be that our study enrolled a large number of SMS‐naive patients, as well as more than 2000 men, a group which is typically under‐represented in many studies focusing on SMS.

Third, despite evidence that some SMS interventions carried out online or by phone are effective, most people prefer face‐to‐face SMS (at GP practice or at home). There are, however, very few SMS interventions delivered in the recipient's own homes, and they are expensive for health care systems. At the same time, there is a lot of ongoing work developing Web‐based SMS interventions. Some of these are developed for very practical reasons such as help individuals with rare conditions or to help the system reduce health care costs. Unfortunately, most of these health apps currently on the market have not been robustly evaluated. Future evaluations of these eHealth SMS interventions may want to consider building on our findings, bearing in mind that these interventions are more likely to be appealing to males with a single LTC in its early stages.

Fourth, it is worth contextualizing our finding with the current level of interest in SMS interventions. Over three quarters of respondents in our survey (approximately 78%) stated that they would use SMS which might fit in with ideas of patient activation. Our analysis, however, could not find systematic differences in PAM levels (or scores) between those who said that they would use the chosen SMS option and those who did not. This may be due to the ceiling effect and other methodological issues associated with the measure itself.^47^ By choosing none of the SMS options offered to them, respondents are not necessarily indicating that they are uninterested in SMS. Rather it suggests that their preference structure included variables that were unaccounted for by the DCE task question under consideration. Future work will need to unveil these participation triggers.

Our findings on the low acceptability of mode of delivery methods typically considered to be cost‐effective (ie, online, non–health professional, group based) is striking and important. There are very few head‐to‐head comparisons of high‐cost versus low‐cost SMS interventions, and the assumption that reducing direct costs will lead to cost‐effectiveness remains untested. It is likely that, where feasible, expanding investment in SMS interventions that meet patients’ preferences, would generate better longer‐term outcomes, resulting in a more cost‐effective provision. Finally, our study generated important evidence that can be used to design, evaluate, and provide value for money person‐centered SMS interventions for people with LTCs.

There are however some important differences between the UK and US health care systems and culture that may affect the transferability of our results from one country to another. The chief contextual differences between the UK and United States are that the UK has universal health care coverage funded by general taxation, while the United States has an insurance based system with 10% of the nonelderly population uninsured.^48^ The United States spends much more of its GDP on health care than the UK (17.2% vs 9.7% in 2016, according to OECD Health figures), and in the UK, secondary health care is usually accessed via primary care services whereas in the United States patients may access specialist secondary care services directly. All of these factors may influence people's preferences around self‐management interventions, for example, self‐management interventions may be more popular in people with long‐term conditions who have no health insurance or who are underinsured. In this sense, UK patients are less used to having to make choices about health care provision, where primary care provision may be their initial “anchor” in terms of expectations. These differences may play an important role in influencing what people prefer in self‐management interventions, and of course, the preferences they may have toward specific SMS services. Future work could focus on testing the external validity of our findings to the US health care context, extending the methodology and data collection to include factors that may be able to explain differences between health care delivery system and culture that may influence choice. Further research should also focus on how to manage the tension between “what people want,” and “what is likely to be feasible” both in terms of implementation and funding. Managing this tension might involve a number of responses, such as understanding people's preferences and using incentive mechanisms to inform their choices, or adopting a stepped care or precision approach to target people better, for example, try to manage as many people as possible with more affordable models (within the set of the feasible options) and having other options for those who are not likely to benefit from the “mainstream” offers.

## Supporting information

Author Matrix

Supplementary Material
