# Supplementary material for: People’s preferences for self‐management support
Source: Health Serv Res. 2021 Feb 25;57(1):91–101. doi: 10.1111/1475-6773.13635 (PMC8763292; doi:10.1111/1475-6773.13635)
Supplement: Supplementary file 2 — Supplementary Material [file HESR-57-91-s002.docx]

**People’s preferences for self-management support: a mixed methods study.**

**Supplementary material**

*Box 1A:*

*Target audience.* Refers to the characteristics of the intended audience for an activity (or training) that is designed to help individuals live better with their long‑term condition (i.e. SMS intervention). For example, this concept considers whether the SMS intervention was looking at one‑to‑one vs. group activity; whether interventions may be involving a group of people with a common long‑term condition vs. a group of people with different conditions; and whether it involved a same gender group people vs. mixed gender group.

*Targeted area of support.* Addresses the ability of SMS interventions to support individuals to improve particular aspects of their self‑management skills and may include areas such as medical knowledge, skills, lifestyle, well‑being, or self‑efficacy, confidence, etc.

*Focus of the SMS intervention*. Comprises five different key components: design of the intervention, technical knowledge and skills, information provision, supporting and promoting change, and safety netting.

*Interaction.* Considers the type of person with whom the patient interacts as part of a SMS intervention (e.g. doctor, non‑healthcare professional) and the main trigger for the interaction.

*Mode of delivery.* Refers to a variety of ways in which individuals could interact with the agent providing them the support to help them live better with their long term condition. For example: face-to-face; over the telephone; paper-based; electronic (e.g. videos, e-mail, text message, internet, etc.).

*Practical issues.* Practical issues covered aspects such as the setting for the SMS intervention, time investment required for the patient, travel distance to receive support (when SMS does not take place at home) and frequency of contact.

**Table 1A: Attributes and attribute levels of SMS interventions**

| **Attribute** | **Attribute-levels** | **Description** |
| --- | --- | --- |
| Main Aim | Improve medical knowledge and skills  Improve my lifestyle and wellbeing  Improve my confidence  Improve my motivation | Primary aim of the SMS activity |
| Format | In person  Telephone  On-line  On paper  Smart phone apps | The way the SMS activity is delivered |
| Who delivers it | Consultant in the condition  Specialized nurse in the condition  GP doctor  GP nurse  Health trainer  Lay person | Characterization of the person delivering the SMS activity based on qualifications and training in healthcare |
| Where it happens | GP  Hospitals  Home  Community | Setting where the SMS takes place |
| Type of interaction | Individual  Group | Number of participants |
| Availability | On demand  Scheduled | Degree of flexibility to access a SMS activity |
| How often it happens | Daily  Weekly  Monthly | This refers to how frequently a SMS activity takes place |
| Contact time | Less than 30 mins  Between 30 mins and 1 hour  More than an hour | Amount of time people would set aside to engage with a SMS activity |
| Travel time | No travel  Less than 30 min  Between 30 mins and 1 hour  More than an hour | If SMS cannot be done at home, this refers to travel time to where the activity takes place |
| Style of interaction | Tells me what to do  Discusses my options with me | Communication style of the person delivering the SMS activity |
| Who endorses it | NHS organizations  Charitable/patient organization  For profit organizations/individual | Refers to the person/institution/ organization that designed and/or is responsible for the content of a SMS activity |

**Table 2A: Distribution of long-term conditions in the final sample**

| **Condition** | **In the sample** | **Expected** |
| --- | --- | --- |
| Chronic Pain | 21% | 19% |
| Asthma | 12% | 8% |
| Chronic obstructive pulmonary disease (COPD) | 6% | 2% |
| Atrial Fibrillation | 2% | 2% |
| Cancer | 2% | 2% |
| Hypertension (high blood pressure) | 19% | 18% |
| Coronary Heart Disease | 3% | 5% |
| Heart Failure | 1% | 1% |
| Hypothyroidism (underactive thyroid) | 5% | 4% |
| Long standing feelings of anxiety and depression | 9% | 12% |
| Peripheral Artery Disease | 0% | 13% |
| Stroke or Transient Ischaemic Attacks (TIAs) | 2% | 2% |
| Chronic (or long-standing) Kidney Disease (CKD) | 2% | 5% |
| Diabetes | 12% | 6% |
| Epilepsy (fits and seizures) | 1% | 1% |
| Obesity | 3% | 0% |
| Total | 100% | 100% |

**Table 3A: Distribution of most burdensome reported long-term condition**

**Condition People with co-morbidity**

N (%)

Chronic Pain 740 (36)

Asthma 104 (5)

Chronic obstructive pulmonary disease (COPD) 118 (6)

Atrial Fibrillation 67 (3)

Cancer 49 (2)

Hypertension (high blood pressure) 133 (6)

Coronary Heart Disease 66 (3)

Heart Failure 37 (2)

Hypothyroidism (underactive thyroid) 46 (2)

Longstanding feelings of anxiety and depression 264 (13)

Peripheral Artery Disease 20 (1)

Stroke or Transient Ischaemic Attacks (TIAs) 26 (1)

Chronic (or long-standing) Kidney Disease (CKD) 15 (1)

Diabetes 277 (13)

Epilepsy (fits and seizures) 13 (1)

Obesity 100 (5)

**Total 2075 100**

**Table 4A: Fifteen most prevalent long-term conditions by gender in the UK**

|  | Overall | Male | Female |
| --- | --- | --- | --- |
| Hypertension (high blood pressure) | 0.18 | 0.49 | 0.51 |
| Long-standing feelings of anxiety and depression | 0.12 | 0.29 | 0.71 |
| Asthma | 0.08 | 0.41 | 0.59 |
| Diabetes | 0.06 | 0.47 | 0.53 |
| Coronary Heart Disease | 0.05 | 0.59 | 0.41 |
| Chronic (or long-standing) Kidney disease (CKD) | 0.05 | 0.40 | 0.60 |
| Hypothyroidism (underactive thyroid) | 0.04 | 0.47 | 0.53 |
| Stroke or Transient Ischaemic Attacks (TIAs) | 0.02 | 0.61 | 0.39 |
| Chronic obstructive pulmonary disease (COPD) | 0.02 | 0.59 | 0.41 |
| Cancer | 0.02 | 0.45 | 0.55 |
| Atrial Fibrillation | 0.02 | 0.56 | 0.44 |
| Heart Failure | 0.01 | 0.56 | 0.44 |
| Epilepsy (fits and seizures) | 0.01 | 0.77 | 0.23 |
| Chronic pain | 0.19 | 0.46 | 0.54 |
| Peripheral artery disease | 0.13 | 0.53 | 0.47 |

Figures are %

**Table 5A: Final classification of generic components and dimensions of self-management interventions**

| **Broad concept** | **Key component** | **Description** | **Attributes** | **Levels** |
| --- | --- | --- | --- | --- |
| Target Audience | Target Audience | Refers to the characteristics of the intended audience for an activity (or training) that is designed to help individuals live better with their long‑term condition (i.e. SMS intervention). | Type of interaction | Individual based  Group level |
| Focus of | Design of SMS intervention | Refers to the style of communication and the level of tailoring to audience-specific characteristics and value of an activity (or training) designed to help individuals to live better with their LTC | Its style | Discuss my options and make a decision for me  Discuss my options and lets me chose |
|  |  |  |  |  |
| Interactions | The person a patient interacts with | Considers the person with whom the patient interacts when receiving the SMS intervention. | Who leads it | Doctor  Nurse  Other healthcare professional  Non-healthcare professional |
|  |  |  |  |  |
| Mode of delivery | Mode of delivery | Refers to who people would prefer to interact with a person who is providing support to help them live better with their LTC. | Degree of  human contact | Face to face  Over the phone or online  On your own |
|  |  |  |  |  |
| Practical issues | Setting | Refers to where people would prefer the activity (or training) designed to help them live better with their LTC took place. | Where it happens | General Practice clinic  Hospital  Home  Community centre |
|  |  |  |  |  |
|  | Time investment | Refers to how much time people would put aside to gain the knowledge, confidence and ability required to look after their LTC | How long it takes | Less than 30 minutes  Between 30 and 60 minutes  More than 60 minutes |
|  |  |  |  |  |
|  | Frequency of contact | Refers to how frequently people would be prepared to engage with an activity (or training) designed to help them live better with their LTC. | How often it happens | Weekly  Monthly  Every three months  Annually |

*SMS=self-management support; LTC=Long-term health condition;*

**
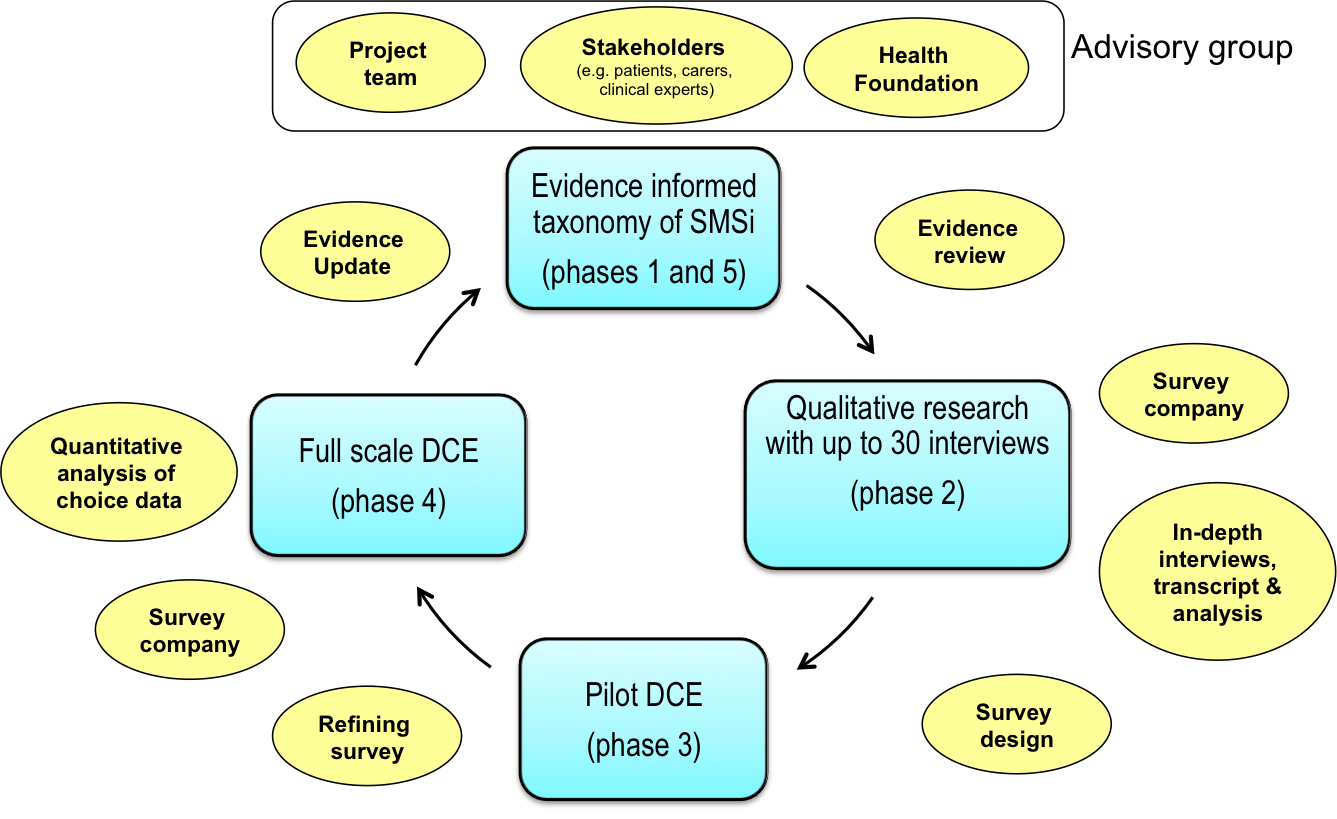
**

**Figure 1A: A visual representation of our research framework**

**Figure 2A. Illustration of a choice-based question included in the final DCE task**

**PART 1**

**HOW DO YOU PREFER TO SELF-MANAGE YOUR CONDITION(S)?**

If these support options are provided to self-manage your condition(s) now, which one would you prefer **the most?**

If you have more than one condition, please answer the following question considering the condition that has **the greatest burden** for you in terms of managing day-to-day activities.

|  | **Hypothetical alternative SMS options** | |
| --- | --- | --- |
| **Attributes** | **Support 1**  **(attribute levels)** | **Support 2**  **(attribute levels)** |
| **How long it takes** | Less than 30 minutes | Less than 30 minutes |
| **How often it happens** | Daily | Weekly |
| **Who leads it** | Lay Person | Lay Person |
| **Its style** | On your own | Face-to-face |
| **Degree of human contact** | Discusses your options  and lets you decide | Discusses your options  and lets you decide |
| Choose ONE by clicking the button |  |  |

**Do you think you would use the option you selected?**

|  | **Yes** |  |
| --- | --- | --- |
|  | **No (please explain why)** |  |
|  | **I don’t know** |  |
